# Convergent evolution of a genomic rearrangement may explain cancer resistance in hystrico- and sciuromorpha rodents

**DOI:** 10.1038/s41514-021-00072-9

**Published:** 2021-09-01

**Authors:** Yachna Jain, Keerthivasan Raanin Chandradoss, Anjoom A. V., Jui Bhattacharya, Mohan Lal, Meenakshi Bagadia, Harpreet Singh, Kuljeet Singh Sandhu

**Affiliations:** Department of Biological Sciences, Indian Institute of Science Education and Research (IISER) - Mohali, SAS Nagar, 140306 Punjab India

**Keywords:** Cancer, Genome, Cancer

## Abstract

The rodents of hystricomorpha and sciuromorpha suborders exhibit remarkably lower incidence of cancer. The underlying genetic basis remains obscure. We report a convergent evolutionary split of human 3p21.31, a locus hosting a large number of tumour-suppressor genes (TSGs) and frequently deleted in several tumour types, in hystrico- and sciuromorphs. Analysis of 34 vertebrate genomes revealed that the synteny of 3p21.31 cluster is functionally and evolutionarily constrained in most placental mammals, but exhibit large genomic interruptions independently in hystricomorphs and sciuromorphs, owing to relaxation of underlying constraints. Hystrico- and sciuromorphs, therefore, escape from pro-tumorigenic co-deletion of several TSGs in *cis*. The split 3p21.31 sub-clusters gained proximity to proto-oncogene clusters from elsewhere, which might further nullify pro-tumorigenic impact of copy number variations due to co-deletion or co-amplification of genes with opposing effects. The split of 3p21.31 locus coincided with the accelerated rate of its gene expression and the body mass evolution of ancestral hystrico- and sciuromorphs. The genes near breakpoints were associated with the traits specific to hystrico- and sciuromorphs, implying adaptive significance. We conclude that the convergently evolved chromosomal interruptions of evolutionarily constrained 3p21.31 cluster might have impacted evolution of cancer resistance, body mass variation and ecological adaptations in hystrico- and sciuromorphs.

## Introduction

Unlike myomorphs (mouse, rat, etc.), hystricomorphs (naked-mole rat, capybara, etc.) and sciuromorphs (squirrels, marmots, etc.) exhibit extraordinary diversity in body mass, longevity and ecological habitat^1–12^. One of the common traits of rodents of hystrico- and sciuromorpha suborders, which gained considerable attention recently, is their apparent resistance to cancer^13–16^. It has been proposed that the large body mass and long lifespan are the evolutionary forces that innovate cancer resistance in mammals^13,16,17^. Accelerated cell proliferation to gain large body mass might lead to cancer^18–20^. To minimise the risk of cancer, many rodents with large body mass have evolved with repressed telomerase activity to allow replicative senescence that suppresses cancer^13,16^. Similarly, to have a longer lifespan, a species may need to delay cancer incidence beyond its reproductive age. Long-living mammals, therefore, evolved with efficient cancer-resistance mechanisms^13,16,21,22^. The cancer resistance of hystricomorphs is particularly well-studied in naked-mole rat (NMR). Tian et al.^14^ has shown that NMRs contain high-molecular-mass Hyaluronan (HMM-HA), a component of the extracellular matrix, unlike other mammals which have low-molecular-mass HA (LMM-HA). This was presumably an evolutionary selection for highly elastic skin adapted to the underground habitat. Interaction of HMM-HA with the CD44 receptor induces p16, which in turn mediates early contact inhibition, an anticancer mechanism that arrests cell division when cells reach a certain density, in NMR^14^. HMM-HA also inhibits the process of metastasis^23,24^. The genome of NMR exhibits remarkable epigenetic stability and efficient DNA repair when compared with that of other mammals, which eventually contributes to cancer resistance^25,26^. Capybara, the largest extant rodent, exhibits cancer resistance through the expansion of genes associated with T-cell-mediated tumour suppression^27^. The Kurloff cells in guinea pig are shown to have anti-leukemic activities^28^. Squirrels also exhibit cancer resistance despite having high telomerase activity^13^. However, the underlying mechanisms are not known. Among other cancer-resistant mammals, genomes of elephants and whales have shown expansions in the copy number of tumour-suppressor genes^29,30^. Bats gained cancer resistance through an efficient genome maintenance mechanism^31^. These observations imply that cancer resistance had evolved independently in phylogenetically distant mammals and that multiple mechanisms can converge to endow cancer resistance to a species.

Copy number variation (CNV) is one of the common mechanisms leading to the gain or loss of relative abundance of proto-oncogenes (POGs) and tumour-suppressor genes (TSGs)^32^. Amplification of one or both copies of a POG can cause cancer^32^. Though both copies of a TSG need to be deleted to remove its tumour-suppressor impact, deletion of either copy predisposes the individuals to cancer^32^. POGs and TSGs can be found in individual gene clusters, likely constrained by concurrent chromatin states and coordinated transcription of co-positioned genes^33^. Clustering of POGs or TSGs accompanies a cost of increased susceptibility to cancer since several oncogenes can be co-amplified causing cancer or several tumour-suppressor genes can be co-deleted to predispose the individuals to cancer^34,35^. Examples include co-amplification of POGs at 8p11–12^36^, at 14q13.3^37–40^ and co-deletion of TSGs at 8p22^41^ and at 3p21.31^42–44^ regions. On the contrary, many other POGs exhibit linear proximity to TSGs under an evolutionary constraint to nullify tumorigenic effect through co-amplification or co-deletion of oncogenes and tumour-suppressor genes^45^. We hypothesise that certain genomic rearrangements of POG and TSG clusters can endow cancer resistance to some mammals. Indeed, the widespread importance of genomic rearrangements in various ecological and evolutionary processes has been recognised^46^. Inversions alone can promote speciation either by generating large genetic barriers needed for reproductive isolation or by suppressing recombination to protect co-adapted alleles^47–49^. Apart from their role in speciation events^50^, genomic rearrangements are non-trivially linked to divergence of gene expression and of phenotypes^51,52^. It is, therefore, tenable to assess the relative genome organisation of cancer susceptible and cancer-resistant mammalian species in order to explore mechanisms of cancer resistance. Through analysis of 31 placental mammalian and 3 non-mammalian vertebrate genomes, we showed that TSG-enriched 3p21.31 locus of human was interrupted by large genomic inserts in hystrico- and sciuromorphs. The interruptions of the 3p21.31 locus were independent and did not share breakpoints in hystrico- and sciuromorphs. The locus remained syntenic in myomorphs as well as in other mammals. 3p21.31 is deleted in most cancers, and with a very high frequency in lung carcinoma, renal cell carcinoma, breast cancer and uterine cervix carcinoma^43^. The evolutionary split of this cluster can, therefore, endow cancer resistance by escaping the co-deletion of several TSGs in *cis*. We further inferred functional and evolutionary implications of our observations in terms of (i) evolutionary constraints that kept the tight synteny of 3p21.31 cluster throughout mammalia, and (ii) relaxed constraints on 3p21.31, body mass evolution and ecological adaptations in hystrico- and sciuromorphs.

## Results

### Genomic rearrangements and cancer resistance in naked-mole rat

**Fig. 1:**
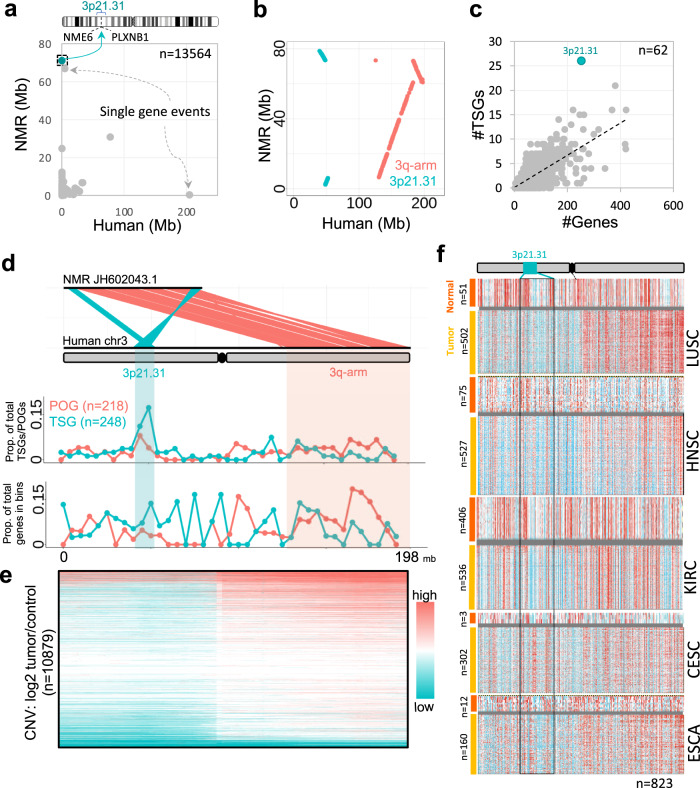
Genomic rearrangements and tumour-suppressor properties of 3p21.31. **a** Scatter plot of all intergenic distances between consecutive genes of human vs. that of corresponding NMR orthologues. The box on the *y* axis highlights the genes (NME6 and PLXNB1) that were proximal in human, but distant in NMR, and marks the large genomic split of 3p21.31 locus. The events of single-gene repositioning are marked by grey arrows. Details of these events are given in Supplementary Fig. 1a, b. **b** Dot plot between human chr3 and NMR JH602043.1 scaffold. Sea-green coloured dots represent 3p21.31 locus, while the 3q arm of chr3 is shown in red. **c** Number of tumour-suppressor genes as a function of the total number of genes in each chromosomal band of human genome. The 3p21.31 cluster is marked in sea-green colour. **d** Upper panel: linear view of genomic rearrangements between human chr3 and NMR JH602043.1. Middle and lower panels: density of proto-oncogenes and tumour-suppressor genes along human chr3, normalised by the total number of TSGs and POGs (upper panel), and by the total number of genes in respective chromosomal bands (lower panel). **e** Copy number variations along human chr3 in TCGA Pan-cancer dataset (*n* = 10,879). Each horizontal line represents one instance of CNV, each row represents one patient, and the colour grade scales to the tumour-to-control ratio of genomic DNA in TCGA Pan-Cancer dataset. **f** Gene expression (RNA-seq data normalised to TCGA cohort) of tumour and normal tissues along chromosome 3. Data shown in panels **e** and **f** were directly obtained from UCSC Xena browser. LUSC lung squamous cell carcinoma, HNSC head–neck squamous cell carcinoma, KIRC kidney renal clear cell carcinoma, CESC cervical squamous cell carcinoma and endocervical adenocarcinoma, ESCA oesophageal carcinoma.

We first identified the instances of genomic rearrangements between a cancer susceptible (human) and a cancer-resistant (NMR) mammal (Fig. 1a–c and S1a, b). Since genome assemblies of NMR are available as collections of large scaffolds and not as chromosomes, we only focussed on *cis* (intra-chromosomal) alterations in this study. We plotted intergenic distances between consecutive human genes against the distances between corresponding NMR genes. We focussed on values scoring high either on *x* axis (Human) or on *y* axis (NMR). We observed that the most meaningful changes were associated with human chromosome 3 mapping to NMR scaffold JH602043.1 (Fig. 1a). The highest value along *y* axis (NMR) marked the split of the 3p21.31 locus from a breakpoint flanked by NME6 and PLXNB1 genes. The second highest value along *y* axis involved chromosomal repositioning of only one gene (OSBPL11) and did not involve large genomic rearrangements. This gene was adjacent to a region annotated as ‘assembly exceptions’, implying the possibility of erroneous sequence mapping (Supplementary Fig. 1a). Similarly, the highest value along *x* axis involved repositioning of a single pseudogene OR9H1P, which might have resulted from differential retrotransposition in human and NMR (Supplementary Fig. 1b). Therefore, we ignored the events of single-gene repositioning and focussed only on 3p21.31 split.

Upon closer inspection of human chr3 and NMR JH602043.1 through dot plot, we found that human 3p21.31 locus and 3q arm were engaged in a complex rearrangement in NMR. In particular, human 3p21.31 locus was interrupted by 71 Mb region from 3q arm in NMR (Fig. 1b). We observed the same through analysis of another genome assembly^53^ of NMR (GCA_014060925.1, Supplementary Table 2 ), suggesting that the split of 3p21.31 locus in NMR was not a genome assembly artefact.

The 3p21.31 locus was highly enriched with TSGs (*n* = 25, Fig. 1c, d), while 3q arm was relatively enriched with POGs (Fig. 1d). This was coherent with the reports that 3p21.31 is a dominant tumour-suppressor locus and 3q26 and 3q29 are OncCassettes^54–56^. To further demonstrate tumour suppressing potential of 3p21.31, we browsed TCGA Pan-cancer data using UCSC Xena browser. The map of copy number variations (CNVs) confirmed that human 3p-arm exhibited more instances of deletions and, on the contrary, 3q arm had more of amplifications (Fig. 1e and S1c). Concomitantly, the locus also exhibited a greater frequency of somatic point mutations in TCGA Pan-cancer cohort (Supplementary Fig. 1d). By mapping gene expression data for various tumour types (TCGA cohort), we observed that genes on 3p-arm were mostly downregulated and those on 3q arm were upregulated in tumour samples when compared with normal (Fig. 1f). Clustering of TSGs at 3p21.31 locus allows their co-deletion or co-silencing, and subsequent predisposition to cancer^42–44,57^. The 25 known TSGs of 3p21.31 were split into sub-clusters of 16 and 9 TSGs by a 71 Mb insert from 3q arm in NMR. The extreme distance constraint would not have allowed the co-deletion (or co-silencing) of all TSGs of 3p21.31 in NMR, and, therefore, might have contributed to cancer resistance.

**Fig. 2:**
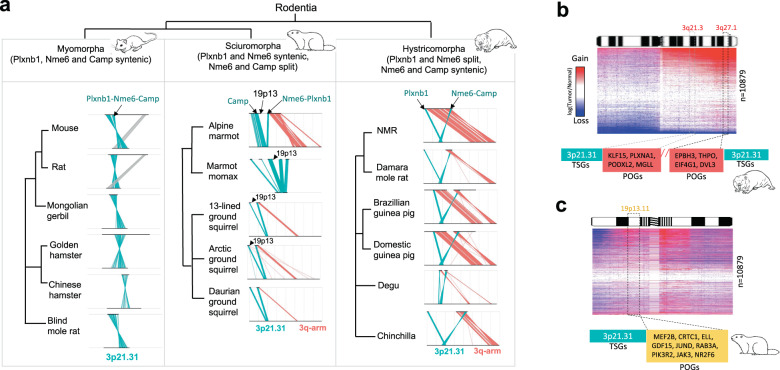
Synteny status of 3p21.31 in major rodent clades. **a** Orthologous genomic position of human 3p21.31 genes in myomorphs, sciuromorphs, and hystricomorphs. The positions of PLXNB1, NME6 and CAMP genes are highlighted. PLXNB1-NME6 breakage marks the 3p21.31 split in hystricomorphs and NME6-CAMP breakage marks the split in sciuromorphs. **b**, **c** Gain of proximities between TSGs from 3p21.31 and the POGs from 3q21.3/3q27.1 in NMR (**b**), and from 19p13.21 in alpine marmot (**c**). Heatmaps represent relative gain and loss of CNVs in the PAN-Cancer cohort normalised to control samples.

We further tested whether the observed rearrangement was specific to NMR. While we observed the synteny of 3p21.31 cluster in myomorphs, all analysed hystricomorphs exhibited the same rearrangement as in NMR, highlighting that the rearrangement occurred in the common ancestor of hystricomorphs (Fig. 2a and Supplementary Table 2 ). Interestingly, sciuromorphs exhibited an independent split of 3p21.31 from a different breakpoint, and involved an insert from 19p13.11 locus (Fig. 2a). While the precise length of this insert could not be determined due to fragmented genome assemblies, it was at least 8 Mb in alpine marmot. Importantly, like in NMR, split 3p21.31 also gained proximity to a known onco-cluster on 19p13.11 in alpine marmot, harbouring at least nine well-known POGs, and frequently amplified in Pan-cancer samples (Fig. 2b, c). The gained proximity to the POGs from 3q arm in hystricomorphs and from 19p13.11 in sciuromorphs might have allowed nullification of tumorigenic effects though co-deletion (or co-silencing) or co-amplification (or co-activation) of co-positioned TSG and POG clusters, as proposed elsewhere^45^.

### Constraints on 3p21.31 locus in placental mammals

**Fig. 3:**
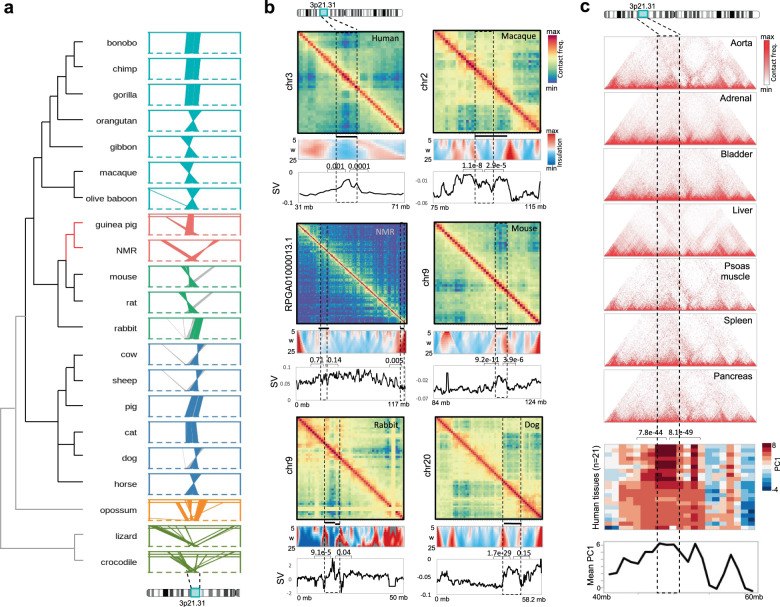
Linear and spatial synteny of 3p21.31 in placental mammals. **a** Location of orthologous regions of human 3p21.31 in primates (bonobo, chimp, gorilla, orangutan, gibbon, macaque and olive baboon), rodents (guinea pig, naked-mole rat, mouse and rat), lagomorph (rabbit), artiodactyls (cow, sheep, pig), canines (dog and cat), perissodactyl (horse), marsupial (opossum) and reptiles (lizard and crocodile). Mouse and rat showed an independent split of a small terminal region of 3p21.31 (grey coloured). This region was outside the tumour-suppressor cluster of 25 genes. **b** Hi-C contact maps (1 Mb resolution), domainograms of insulation scores (200 kb resolution), and leading singular vectors (SV, 200 kb resolution) of 3p21.31 and adjacent regions in the liver of human, macaque, NMR, mouse, dog and rabbit. Black coloured bars above domainograms indicate TAD(s) overlapping with 3p21.31. *P* values were calculated for the difference of values within 3p21.31 and the adjacent regions of the same length using two-tailed unpaired *t* tests. **c** Hi-C contact maps (40 kb resolution), compartment scores (leading principal component as per Schmitt et al.^130^) of 3p21.31 and adjacent regions across different human tissues. *P* values for the difference of values within 3p21.31 and the adjacent regions of the same length were calculated using two-tailed unpaired *t* tests. Effect sizes: 3.35, 2.22 (human); 1.90, 1.27 (monkey); 2.69, 1.59 (mouse); 0.17, 0.74, 1.32 (NMR); 0.91, 0.44 (rabbit); 4.13, 0.28 (dog).

We further extended our analyses to other placental mammals, reptiles and a marsupial. The analysis suggested that 3p21.31 remained syntenic in placental mammals, but not in other terrestrial vertebrates (Fig. 3a), implying that the common ancestor of placental mammals gained the clustering of genes at 3p21.31 locus. We suggest that the selection for terrestrial and mammalian adaptations might underlie the observed gain of pronounced gene clustering at 3p21.31 in placental mammals, as discussed in detail in the ‘Discussion’ section.

The 3p21.31 locus was spatially converged into one complex in human, rhesus macaque, mouse, dog and rabbit as revealed through the analysis of chromosome conformation datasets (Fig. 3b, c). Interestingly, 3p21.31 locus showed unique spatial seclusion from the rest of the chromosome 3 as inferred from strikingly low inter-loci, and high intra-locus contact frequencies in Hi-C contact maps across different tissues and species (Fig. 3b, c). The spatial seclusion of 3p21.31 locus was also confirmed through insulation scores, plotted as domainograms and the lead singular vectors (SV) of normalised Hi-C contact matrices. The 3p21.31 locus exhibited strong insulation signals at borders and converged to a compartment independent from the rest of the chromosome as marked by higher values of singular vector (Fig. 3b, c and Supplementary Fig. 2). Interestingly, the only other loci that interacted with 3p21.31 on chr3 were the regions marking the human NMR breakpoints on the chr3q arm (Supplementary Fig. 2), implying that the spatial proximity of these loci in the ancestral genome may have mediated the genomic rearrangements, as witnessed elsewhere^58^. In NMR, as expected, the split 3p21.31 sub-clusters were positioned in separate and distant topological complexes. However, interestingly, one of the split 3p21.31 clusters (left one in Fig. 3b—NMR) was not strongly insulated, and seemingly had spatial mixing with 3q. Since CNVs often span within the range of topological complexes, the above observation may support the possibility of co-deletion or co-amplification of split TSG cluster together with the adjacent POG cluster from 3q, which may nullify pro-tumorigenic potential of CNVs spanning genes of opposing effects, as mentioned earlier.

**Fig. 4:**
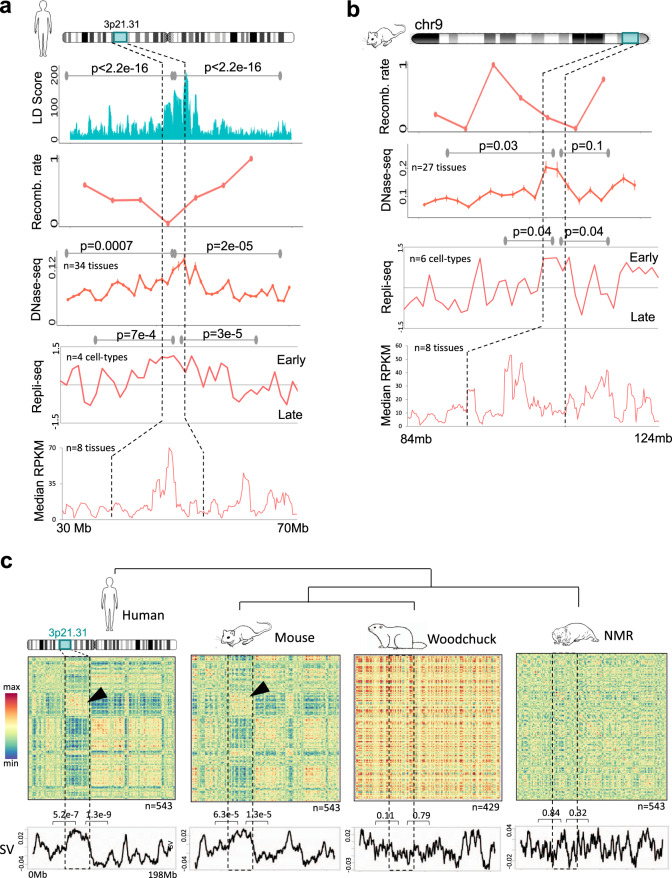
Genetic and epigenetic attributes of 3p21.31 in human and mouse. **a**, **b** Linkage disequilibrium (LD) score, recombination rate, chromatin accessibility (across 34 human and 27 mouse tissues), replication timing (across four human and six mouse cell types) and the median expression levels of genes (across eight tissues) in human (**a**) and in mouse (**b**). *P* values for the comparison of values in the 3p21.31 locus and in the adjacent regions were calculated using two-sided Mann–Whitney *U* tests. Error bars represent 95% confidence intervals. Effect sizes: 0.25 and 0.29 for LD score; 0.63 and 0.41 for DNase-seq (human); 0.66 and 0.16 for DNase-seq (mouse); 0.99 and 1.28 for Repli-seq (human); 0.66 and 1.2 for Repli-seq (mouse). **c** Co-expression heatmaps of genes from human chromosome 3, and the corresponding orthologues in mouse, woodchuck and naked-mole rat. Below each heatmap is the leading singular vector (SV) of co-expression matrix. High overall SV values at 3p21.31 in human and mouse signify its secluded transcriptional co-regulation, which was largely independent from rest of the chr3. *P* values were calculated using two-tailed unpaired *t*-test using the same approach as in Fig. 3b. Effect sizes: 0.82, 1.01 (human); 0.64, 0.71 (mouse); 0.26, 0.04 (woodchuck); 0.02, 0.15 (NMR).

The locus exhibited constitutive early replication, low recombination rate, greater linkage, constitutive open chromatin and higher gene expression (median across eight tissues) in human and mouse, implying selection against expression noise, mutations, and allelic recombination that can break the combinations of beneficial and coordinating tumour-suppressor alleles (Fig. 4a, b). Since co-expression of genes is postulated as one of the potent constraints in the evolution of gene clusters^59^, we calculated the co-expression matrices using normalised expression data of several different tissues of human, mouse, NMR and woodchuck (‘Methods’). The genes within 3p21.31 were co-expressed in human and mouse, but not in NMR and woodchuck, again in a manner that was independent of the rest of chromosome 3 (Fig. 4c). The linearly and spatially constrained co-regulation of genes within 3p21.31 in mammals may imply selection on coordinated maintenance of transcriptional states of genes. Interestingly, some genes at 3p21.31 locus are known to interact at protein levels^60^. Their co-expression, therefore, may balance the protein concentrations of interacting partners. Further, the genomic rearrangements of 3p21.31, coincided with the diverged co-expression landscape of genes in hystrico- and sciuromorphs, suggesting the possibility that the relaxed constraint on co-expression might have allowed 3p21.31 to split. The genomic rearrangement of 3p21.3, in turn, may have further facilitated the expression divergence of 3p21.31 genes through the altered genomic neighbourhood.

Overall, the conserved synteny in a wide range of phylogenetically distant placental mammals and the genomic attributes thereof highlight the functional constraints on 3p21.31 locus. Indeed, Makino et al. captured the 3p21.31 locus among the 37 highly conserved interacting gene clusters, which exhibit protein–protein interactions of their gene products, implying the constraints on the linkage of favourable alleles^60^.

### Divergence of 3p21.31 in hystrico- and sciuromorphs

**Fig. 5:**
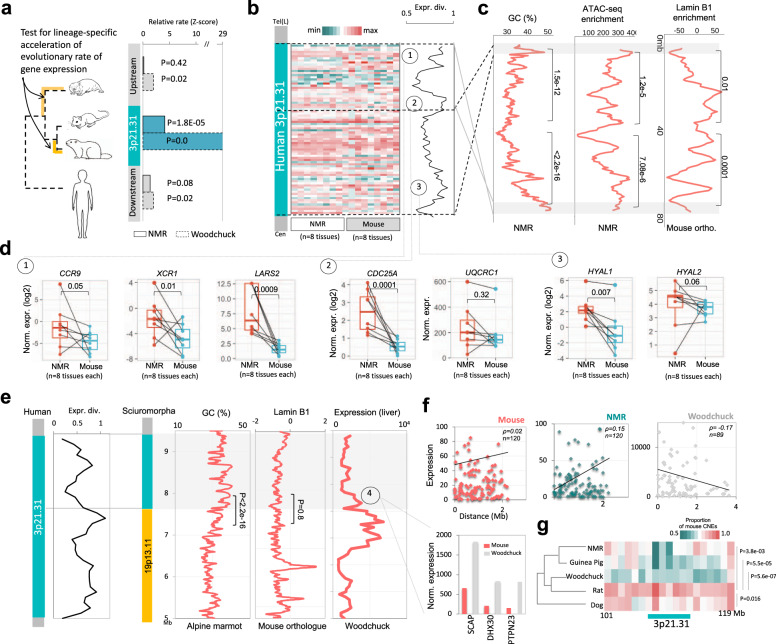
Hystrico- and sciuromorph-specific relaxation of functional constraints on 3p21.31. **a** Rate of gene expression evolution in NMR and woodchuck, considering mouse as control and human as an outgroup. Expression rates are presented as *Z* score and the *P* values are from *Z* test. **b** Heatmap: quantile-normalised expression data of 3p21.31 genes in the skin, thymus, ovary, kidney, adrenal, heart, testes and liver (in that order) of mouse and of NMR. Line plot: mouse-to-NMR expression divergence of genes. **c** GC content, chromatin accessibility (ATAC-seq enrichment in NMR skin fibroblasts), and ChIP-Seq LaminB1 enrichment (mouse fibroblasts) along NMR scaffold JH602043.1. *P* values were calculated using two-sided Mann–Whitney *U* tests. Effect sizes: 0.54 and 0.73 for GC content; 0.17 and 0.17 for ATAC-seq; 0.073 and 0.18 for LaminB1 enrichment. **d** Quantile-normalised gene expression values of genes (for the same tissues as in **b**) located towards the breakpoint regions that are highlighted in the line plot of 3p21.31 expression divergence. The matching tissues of the mouse and NMR are marked by connecting lines between paired boxes. *P* values were calculated using two-sided Mann–Whitney *U* tests. Boxplot annotation: centre line, median; box limits, upper and lower quartiles; whiskers, 1.5× interquartile range; points, outliers. Effect sizes: 0.19, 0.28, 0.70, 0.70, 0.06, 0.39, and 0.15 from left to right. A comparison of normalised expression values of mouse and woodchuck genes near the breakpoint region-4 is shown in the barplot. **e** Left panel: gene-wise expression divergence between mouse and woodchuck along 3p21.31 locus. Right panel: GC content (Alpine marmot), LaminB1 enrichment (in mouse orthologous regions), and gene expression (woodchuck liver) across the rearranged locus involving part of 3p21.31 and 19p13.11 loci. *P* values were calculated using two-sided Mann–Whitney *U* tests. Effect sizes: 0.54 for GC content, 7.9e-6 for LaminB1 enrichment. **f** Expression of 3p21.31 genes in mouse, NMR and woodchuck as a function of distance from the nearest breakpoint. Mean expression levels across tissues were plotted. Effect sizes: Pearson’s correlation coefficient (ρ) are mentioned in the plots. **g** Proportion of mouse-conserved mammalian CNEs present in NMR, woodchuck, guinea pig, rat and dog. *P* values were calculated using two-tailed *t* tests between rat and other species. Effect sizes: 1.91, 3.24, 5.12, 1.65 for NMR–rat, GP–rat, Woodchuck–rat and dog–rat comparisons respectively.

We tested if the gene expression within 3p21.31 evolved more rapidly in NMR and woodchuck as compared to mouse. We assumed Ornstein–Uhlenbeck (OU) model to calculate expression distances among species and assessed relative evolutionary rates of expression divergence in NMR and woodchuck against that of mouse, taking human as an outgroup. We used the method given by Ruan et al.^61^. Locus 3p21.31 exhibited a significantly greater evolutionary rate of expression divergence in NMR and woodchuck from that of mouse (*P*_Woodchuck_ = 0.0, *P*_NMR_ = 1.8e–05), while adjacent regions showed insignificant or marginally significant rates (*P*_Woodchuck_ = 0.02, 0.02, *P*_NMR_ = 0.42, 0.08; Fig. 5a). The expression divergence was particularly higher towards the DNA breakpoints, suggesting the possibility of chromosomal position effect following genomic rearrangements (Fig. 5b). Indeed, the split 3p21.31 sub-clusters gained proximities to AT-rich regions, which exhibited lower overall ATAC-seq signal in NMR, implying a relatively heterochromatic environment in-between split sub-clusters of 3p21.31 (*P*_GC_ = 1.5e–12, <2.2e–16, *P*_ATAC-seq_ = 1.2e–5, 7e-6; Fig. 5c). This was reinforced by the significant enrichment of lamina-associated domains (LADs) in the 3q orthologue in mouse (*P*_LAD_ = 0.01, 0.0001; Fig. 5c). These observations suggested that the genomic interruption of 3p21.31 in hystricomorphs might have caused the expression divergence of genes through gained proximity to relatively impermissible chromatin neighbourhood. Indeed, we observed relative downregulation of genes located near the breakpoints in NMR, but not in mouse (Fig. 5d–f). In particular, the presence and the relative downregulation of hyaluronidases (HYALs) and chemokine receptors (CCRs), located near the terminal breakpoints of 3p21.31, was important given their association with cancer resistance and other hystricomorph-specific phenotypes like elastic skin, lack of inflammatory response, etc. (Fig. 5d). Among other downregulated genes were LARS2, a mitochondrial Leucyl t-RNA synthetase gene, known to have an association with hearing loss in human^62^, CDC25A associated with vision^63,64^, and UQCRC1 associated with oxidative phosphorylation and known to be downregulated in hypoxic conditions^65,66^ (Fig. 5d). Sciuromorph orthologue of 3p21.31 also exhibited expression divergence near the breakpoints. Unlike the 3q-insert in hystricomorphs, 19p13.11 insert in sciuromorph had relatively less contrasting GC content to that of 3p21.31, was depleted in LaminB1 enrichment, and had high expression levels of genes (Fig. 5e). The 3p21.31 genes near the breakpoint had higher expression levels in woodchuck when compared with the mouse. These genes include SCAP, DHX30, and PTPN23. The SCAP gene functions in cholesterol and lipid homoeostasis^67^, DHX30 regulates the ribosome assembly^68^ and PTPN23 is a protein tyrosine phosphatase^69^ (Fig. 5e, f). All three functions, namely lipid homoeostasis, ribosome assembly and protein (de)phosphorylation have diverged regulation in sciuromorpha due to periodic hibernations^70,71^. These observations together supported the implications of chromosomal position effects associated with 3p21.31 rearrangement on expression and phenotypic divergence of hystrico- and sciuromorphs.

It, however, remains counterintuitive that the expression of TSGs also diverged in the cancer-resistant rodent clades. Particularly, the relative downregulation of 3p21.31 genes, including TSGs, in NMR was surprising. We propose the following explanations: (i) TSGs are growth suppressors, and their expression divergence may relate to body mass diversification in the cancer-resistant rodent clades, as explored in further sections; (ii) complementary cancer-resistance mechanisms like multiple paralogous copies of TSGs^72^, the longer half-life of TSGs^73^, low mutation rate^74^, stable epigenome^25^, efficient DNA repair^26^, accumulation of high-molecular-weight hyaluronan^14^, etc. may have relieved the constraints on expression levels of TSGs at 3p21.31 in hystricomorphs.

Further, it is recognised that conserved non-coding elements (CNEs) impose constraints on the linear clustering of genes^75^. We, therefore, analysed the relative loss of CNEs in 3p21.31 region. We observed that NMR, guinea pig and woodchuck exhibited significant loss of mouse-conserved mammalian CNEs in 3p21.31 when compared with that in rat and dog (an outgroup)(*P*_NMR_ = 3.18e–3, *P*_GP_ = 5.5e–5, *P*_Woodchuck_ = 5.5e7, *P*_Dog_ = 0.016; Fig. 5g). This concomitantly highlighted that the constraints on 3p21.31 synteny were relieved in hystrico- and sciuromorphs.

### Accelerated rates of body mass evolution in hystrico- and sciuromorphs

**Fig. 6:**
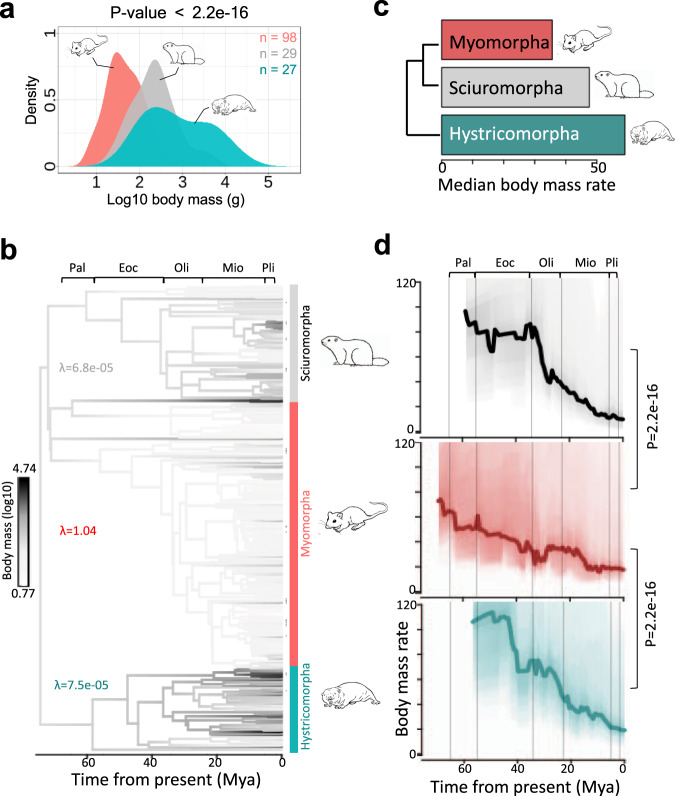
Rate of body mass evolution in rodent clades. **a** Distribution of log10 body mass of myo-, hystrico- and sciuromorphs. *P* value was calculated using Kruskal–Wallis test. **b** Ancestral reconstruction of body mass across phylogenetic tree of 312 rodent species. Pagel’s *λ* values were calculated using ‘phyloSig’ function of ‘phytools’. **c** Median rate of body mass evolution in myo-, hystrico- and sciuromorphs. **d** Rate of body mass evolution in myo-, hystrico- and sciruomorphs as a function time from the present. Body mass rates were calculated using the BAMM model. *P* values were calculated using two-sided Mann–Whitney *U* tests.

**Fig. 7:**
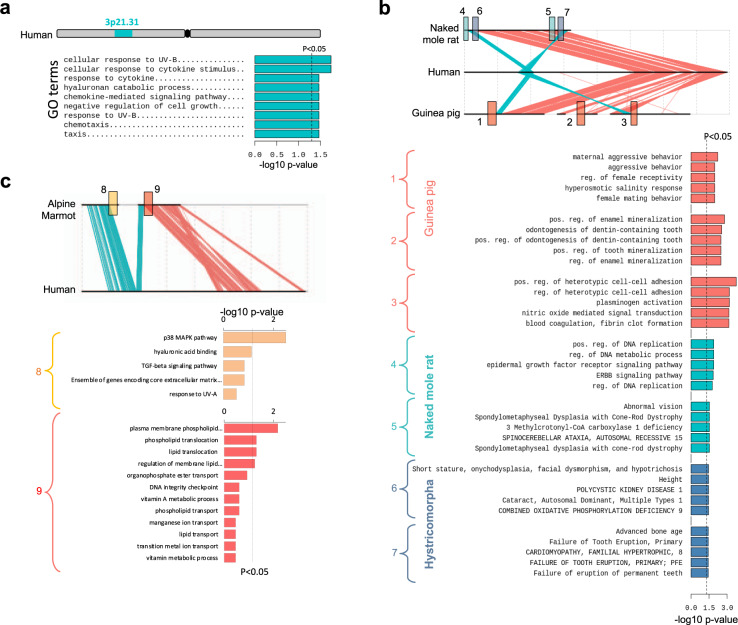
Function of genes located within 3p21.31 and near the breakpoints in hystrico- and sciuromorphs. **a** Enrichment of gene ontology terms in 3p21.31 locus. **b**, **c** Enrichment of gene ontology terms in regions near various breakpoints in NMR, guinea pig, and alpine marmot. The breakpoint regions 1–3 are specific to guinea pig. Regions 4–5 are specific to NMR, 6–7 are common to both the hystricomorphs, and 8–9 are specific to Aalpine marmot. All gene ontology analyses were performed using ToppGene suite with P-value correction through Benjamini–Hochberg method.

Tumour-suppressor genes are negative regulators of cell proliferation^76–78^. We indeed captured the ontology term ‘negative regulation of cell growth’ among 3p21.31 genes (FDR < 0.05, Fig. 7a). We, therefore, tested if the evolutionary rates of body mass variation differed in myomorphs, sciuromorphs and hystricomorphs. We first confirmed that their body mass differed significantly, and the latter two exhibited a greater mean and variance thereof (*P* = 2.2e–16; Fig. 6a, b). Indeed, the diversity of body mass in sciuro- and hystricomorphs was also apparent from the Pagel’s *λ* values of the three clades. Pagel’s *λ* captures the tendency of a trait distribution in a phylogenetic clade to match with the expected distribution under the assumption of the Brownian model of evolution^79^. A *λ* value closer to 1 signifies a higher degree of similarity of trait of a species to its phylogenetically closer, as compared to distant, species. Close to 1 value of Pagels’ *λ* is also sometimes considered as a proxy to higher evolutionary constraints on a trait. Pagels’ *λ* value significantly less than 1 might, but not necessarily, mark a lower evolutionary constraint on a trait. Accordingly, we observed that myomorpha clade had *λ* = 1.04, while sciuromopha and hystricmorpha clades had *λ* = 6.8e–05 and 7.5e = 05, respectively (Fig. 6b).

Through Bayesian Analysis of Macro-evolutionary Mixtures (BAMM)^80^, we calculated phylogenetic rates of body mass (log10) evolution across 312 rodents for 1 million generations. Fig. 6c, d showed significantly higher rates of body mass evolution in sciuro- and hystricomorphs, particularly during Eocene epoch, when compared with that of myomorphs (*P* = 2.2e–12). Indeed, Eocene marked the explosive radiations of mammals that produced the great diversity of mammals known today. The higher rate of body mass evolution in the ancestral sciuromorph and the ancestral hystricomorph also coincided with our observation that the 3p21.31 locus was split into all the analysed hystricomorphs and sciuromorphs. i.e., 3p21.31 locus likely split in the independent common ancestors of sciuromorphs and hystricomorphs. Based on these observations, we infer that the evolutionary constraint on the body mass was relaxed during the speciation of sciuromorpha and hystricomorpha rodents. This can be a consequence of Eocene climate change. The mid-Eocene is marked with emergence of more open habitats like savannah and scarce woodlands, as inferred from phytolith assemblage analyses. The constraints from cellulose fermentation due to dietary shift from browsing to grazing, the population dynamics of competitors and predatory carnivores, etc. might have impacted the rate of body mass evolution^81,82^.

### Split 3p21.31 gained proximity to genes associated with hystrico- and sciuromorpha-specific traits

The genes within 3p21.31 were associated with negative regulation of growth, response to UV-B, response to cytokines/chemokines, hyaluronan catabolism, etc., hinting at the functions that are needed for terrestrial adaptations of most mammals, but are diverged in hystricopmorphs and sciuromorphs ('Discussion' section). To further understand the functional relevance of the observed genomic rearrangements, we tested the involved breakpoint regions, outside 3p21.31, in hystrico- and sciuromorphs for gene ontology enrichments. Genes near breakpoints in hystricomorphs were associated with hystricomorph-specific traits, like dental/enamel mineralisation, mating behaviour, blood coagulation, oxidative phosphorylation, DNA metabolic process, EGFR pathway, vision etc. (FDR < 0.05; Fig. 7b). Most of these terms are tightly linked to the ecological adaptations of hystricomorphs. For example, it is known that the teeth and the dentary organisations are adapted to assist in digging action in naked-mole rat, and grinding action in guinea pig^83–85^. Some hystricomorphs, like guinea pigs, exhibit diverse mating behaviour ranging from monogamy to promiscuity^86^. Naked-mole rats, in contrast, have an organised eusocial-type mating behaviour^7^. Blood coagulation has been proposed to be an adaptive trait of hystricomorphs to efficiently repair the wounds incurred due to digging, fighting, and being preyed^63,87,88^. Oxidative phosphorylation term is associated with hypoxic conditions in underground and semi-aquatic habitats of some of the hystricomorphs^63,89–92^. DNA metabolic process is associated with DNA repair mechanisms and it is well appreciated that some hystricomorphs have efficient DNA repair systems^26,93^. EGFR pathway is associated with wound healing and pain sensitivity or lack thereof^94–96^. The subterranean hystricomorphs are also known to have poor vision^97^, though whether or not loss of vision is adaptive or neutral is arguable. Similarly, 19p13.11 genes and 3q genes that gained proximities to 3p21.31 split sub-cluster in sciuromorphs were associated with MAP kinase pathway, hyaluronan binding, ECM, lipid transport, vitamin A metabolism, telomere maintenance, etc. (Fig. 7c and Supplementary Table 3). These functions have known associations with torpor and hibernation in sciuromorphs. Concerted changes have been observed in the MAP kinase pathway during hibernation, which may relate to stress tolerance in sciuromorphs during hibernation^98^. The cerebral cortex transcriptome of hibernating ground squirrels shows peaked expression of extracellular matrix components, including hyaluronan-binding proteins, laminins and collagens^99^. Dynamic regulation of ECM components may endow plasticity to the hibernating brain. Further, hibernating mammals primarily feed on stored lipids, which are accumulated in bulk during the activity period. Dramatic alterations in fuel utilisation are the hallmarks of hibernation, and explain the enrichment of lipid metabolism and transport-related terms^70^. Similarly, genes associated with vitamin A metabolism and transport are upregulated during hibernation, suggesting their potential role in transducing photoperiod^100^. Hibernating rodents also exhibit seasonal variation in telomere length^101^. In particular, their telomere lengths increase during hibernation, which may slow ageing.

The analyses of gene annotations, therefore, highlighted the adaptive significance of 3p21.31 rearrangement in the evolution of traits specific to hystrico- and sciuromorphs.

## Discussion

Fronicke et al. had earlier proposed the eutherian ancestry of 3p21.31 cluster^102^. The lack of 3p21.31 clustering in marsupial and non-mammalian vertebrates suggested that the clustering was gained in the common ancestor of placental mammals. Remarkable regenerative capabilities of vertebrates like teleosts, reptiles, amphibians and marsupials likely conferred anti-tumorigenic properties to these vertebrates^103,104^. Mammals may have lost regenerative abilities during evolution, possibly as a trade-off between wound healing and tissue regeneration^105^. Being homoeothermic, mammals cannot tolerate excessive bleeding and are likely to die before any significant tissue regeneration^105^. Instead, efficient wound healing became essential in mammals, though at the cost of lost anti-tumorigenic ability^105^. Mammals, therefore, evolved with other mechanisms to select against tumorigenesis. Quality control of tumour-suppressor mechanisms can be one strategy to effectively avoid cancer. Early replication and consistently open chromatin state ensure genetic integrity of tumour-suppressor loci through minimising genetic and epigenetic lesions owing to spatially central localisation in the cell nucleus and ample availability of DNA proofreading and repair enzymes to the early replicating open chromatin^106–111^. An effective way of ensuring these properties is through positional clustering of genes^33,112,113^, as in the case of 3p21.31. The coordinated alteration in chromatin states and expression of genes in 3p21.31 had been observed by others^57^. It has been proposed that the clustering of TSGs evolved to suppress tumours in a coordinated manner^57^. Interestingly, Makino et al.^60^ captured 3p21.31 as one of the highly conserved ‘interacting gene clusters’, which are constrained by the protein–protein interactions of the linearly co-positioned genes^60^. The lower recombination rate of the 3p21.31 locus can be explained by linkage constraint to keep the combination of fitter alleles together. Yeaman^114^ had postulated that the genes important for adaptations to the local environment tend to cluster together in order to keep the combination of fitter alleles given that the adaptation is guided by the change in allelic frequency at multiple loci^114^. On similar lines, we propose that the clustering of genes associated with growth limitation, UV sensitivity and chemokine response at 3p21.31 together might represent the shared adaptation to the terrestrial habitat of mammals (Fig. 7a). The enrichment of the term ‘negative regulation of growth’ might relate to constraints on the body mass of terrestrial mammals by factors like (i) selection against cancer^115^, (ii) increased energetic cost of being endothermic^116^, (iii) energy investment in gestation^117^ and (iv) skeletal and muscular limitation of limbs to support a load of body mass under the influence of gravity^118^. Terrestrial adaptation to protect against UV radiation explains the enrichment of genes associated with the term ‘response to UV radiation’^119,120^ (Fig. 7a). Inflammation and deposition of matrix proteins are the essential steps in wound healing^121,122^. The enrichment of chemokine receptors and matrix proteins (collagen, laminin, etc.) in the 3p21.31 locus may explain the enrichment of ‘response to chemokine stimulus’ term (Fig. 7a). These proposals might largely explain the evolutionary constraints on the synteny of the 3p21.31 cluster in placental mammals.

Quantitative evidence of body mass variations in hystrico- and sciuromorphs is overwhelming^1,123–125^. We showed that the rate of body mass evolution was higher in the ancestral hystricomorph and sciuromorph, which coincided with the ancestral nature of the 3p21.31 split, and the higher expression rate of genes therein. Indeed, the expression divergence of most tumour-suppressor genes, including that of 3p21.31, is associated with growth-related phenotypes^76–78^. It is, therefore, tenable to reconcile that split of 3p21.31 locus and the expression divergence thereof might associate with the body mass divergence in hystrico- and sciuromorphs during Eocene, an epoch known to be associated with the emergence of open grasslands, widespread body mass divergence, and mammalian radiation. There are also evidence that many ancestral rodents, like those of South America, attained body mass in the open grassland due to the absence of competitors and the placental carnivores^81^. These associations imply the following possibilities:

1. Hystrico- and sciuromorphs were released from the evolutionary constraints on the body mass of ancestral rodent, due to the emergence of more open habitat, wider home range, and relative under-representation of competitors and predators. This might have relaxed the functional constraints on 3p21.31 synteny, i.e. the expression levels of growth suppressor genes were allowed to diverge. Figure 6a, b supported the relaxation of body mass constraint in hystrico- and sciuromorphs.
2. The complementary cancer-resistance mechanisms like efficient DNA repair, duplications of TSGs, the longer half-life of TSGs, stable epigenome, etc. might have allowed the relaxation of selection pressure to keep all TSGs of 3p21.31 together for coordinated tumour suppression. Consequently, the split of 3p21.31 was tolerated during the speciation of cancer-resistant rodent clades.
3. Hystrico- and sciuromorphs gained an adaptive advantage of the 3p21.31 split and expression divergence thereof. This was supported by the presence of genes associated with hystrico- and sciuromorpha-specific traits in the genomic regions that gained proximity to split 3p21.31 sub-clusters (Fig. 7 and Supplementary Table 3). The observed genomic rearrangements of the 3p21.31 locus may, therefore, be evolutionarily selected.

**Fig. 8:**
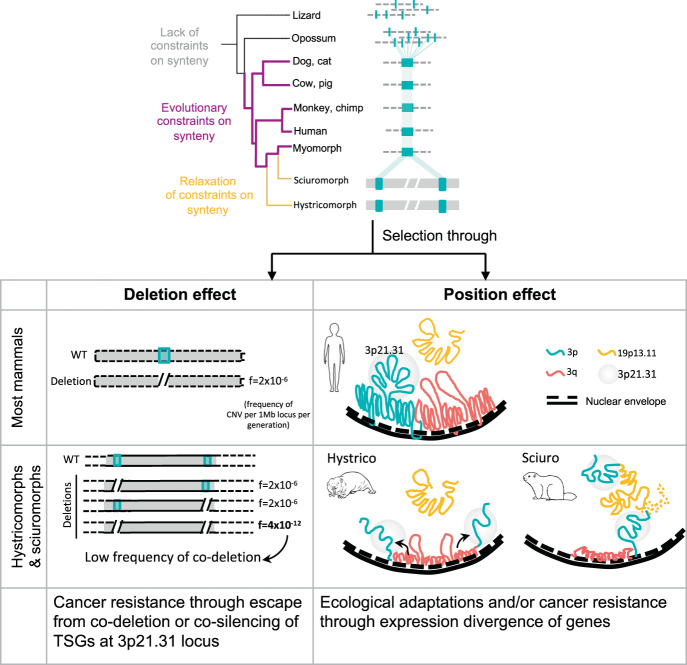
The proposed model for the evolution of cancer resistance in hystrico- and sciuromorphs. The frequency of CNVs per Mb per generation was obtained from Itsara et al.^133^.

It was intriguing that 3p21.31 locus was split from different breakpoints, and had acquired different neighbourhoods in hystrico- and sciuromorphs. The convergent nature of 3p21.31 split in different phylogenetic clades argues strongly against the neutral drift, possibilities of genome assembly artefacts, etc., and instead represents independent ‘biological replicates’ confirming the outcome of an evolutionary experiment and natural selection thereof. Based on our observations, we propose a model for the evolution of cancer resistance in hystrico- and sciuromorph through genomic interruption of 3p21.31 TSG cluster (Fig. 8).

It is also notable that, unlike myomorphs, most hystrico- and sciuromorphs exhibit di-urnality, which strongly associates with high speciation rates in mammals. The large structural variations in genome promotes speciation by reducing the fertility of heterozygotes due to disruptive chromosomal segregations and the meiotic silencing of unpaired chromosomes^126^. Coherently, hystricomorpha exhibits a high rate of karyotype evolution^127,128^ as well as one of the largest shifts towards high speciation rate across mammals. These observations together support the possibility that: (i) the high rate of speciation may itself have innovated unique traits like cancer resistance among others, or conversely, (ii) the relative ‘evolvability’^129^ of lineages, marked by a distinctly higher rate of trait diversification, in this case, may have shaped the dynamics of speciation, further widening the scope our findings.

In summary, we showed that the linear clustering of genes, while having functional and evolutionary significance, might have predisposed mammals to pro-tumorigenic copy number variations. Genomic interruptions of proto-oncogene and tumour-suppressor gene clusters might, therefore, endow cancer resistance to some mammals, like hystricomorphs and sciuromorphs. Convergent evolution of 3p21.31 split further negated the possibility of neutral evolution or technical artefacts and strengthened the underlying evolutionary significance. Altogether, our observations highlight that linear and spatial alterations in genome organisation might guide the evolution of lineage-specific phenotypes.

## Methods

We did not use any statistical method to predetermine sample size. We did not randomise any experiments. We were not blinded to allocations during experiments and outcome assessment. We mentioned the sample sizes and statistical tests wherever applicable. The Source of each dataset is listed in Supplementary Table 1.

### Analysis of genomic rearrangements between NMR and human

We obtained coordinates of orthologous genes of human (hg38) and NMR (hetGla2) from Ensembl biomart (https://www.ensembl.org/biomart/martview). Only one-to-one orthologues were considered to map synteny and genomic rearrangements. We calculated distances between transcription start sites of consecutive gene-pairs of human genome, and scatter-plotted against those of corresponding NMR orthologues using the ‘geom_point’ function of ‘ggplot2’ R-package (https://ggplot2.tidyverse.org/).

### Dot plot

To obtain the dot-plot view of human chr3 and NMR scaffold JH602043.1, we scatter-plotted the gene-TSS coordinates of human chr3 and that of corresponding orthologues in NMR using the ‘geom_point’ function of ‘ggplot2’ in R-package.

### Analysis of cancer-associated properties of human chr3

We obtained the lists of tumour-suppressor genes (TSGs, *n* = 2070) and proto-oncogenes (POGs, *n* = 2511) from CancerMine database (https://bionlp.bcgsc.ca/cancermine/). Genes were binned in 5-mb bins, and the proportions of TSGs and POGs were calculated by: (i) dividing TSG and POG counts by total number of TSG and POGs, respectively, in the genome, and (b) dividing TSG and POG counts by the total number of genes in each bin. For copy number variation analysis, we obtained log2 ratio of tumour-to-normal values of genomic copy numbers across human chr3 from TCGA Pan-Cancer (TCGA-PANCAN) data available at UCSC Xena browser (https://xenabrowser.net/). Similarly, the gene expression data of normal and cancer samples were obtained from ‘IlluminaHiSeq pancan-normalised’ track of Xena browser for various different TCGA cancer types. The heatmaps of log2-transformed values were obtained directly from the Xena browser and colours were transformed to green (#339999) and red (#FF6666) colours using gimp tool (https://www.gimp.org). Somatic point mutation data in TCGA-pancan cohort was also directly obtained from Xena browser.

### Mapping of 3p21.31 locus across other genomes

We obtained the chromosomal coordinates of orthologous genes of human (hg38) in the following species: Alpine marmot (marMar2.1), woodchuck (GCA_901343595.1), 13-lined ground squirrel (SpeTri2.0), arctic ground squirrel (ASM342692v1), daurian ground squirrel (ASM240643v1), mongolian gerbil (MunDraft-v1.0), golden hamster (MesAur1.0), chinese hamster (CriGri_1.0), blind mole rat (S.galili_v1.0), damara mole rat (DMR_v1.0), brazilian guinea pig (CavAp1.0), degu (OctDeg1.0), chinchilla (ChiLan1.0), NMR (hetGla2, GCA_014060925.1), mouse (GRCm38.p6), rat (Rnor_6.0), domestic guinea pig (Cavpor3.0), bonobo (panpan1.1), chimpanzee (Pan_tro_3.0), gorilla (gorGor4), orangutan (PPYG2), gibbon (Nleu_3.0), macaque (Mmul_8.0.1), olive baboon (Panu_3.0), rabbit (OryCun2.0), cow (ARS-UCD1.2), sheep (Oar_v3.1), pig (Sscrofa11.1), cat (Felis_catus_9.0), dog (CanFam3.1), horse (EquCab3.0), opossum (monDom5), lizard (AnoCar2.0), and crocodile (CroPor_comp1) from Ensembl biomart (https://www.ensembl.org/biomart/martview/). One-to-many orthologues, and scaffolds with <5 genes were removed. To plot the synteny maps, we paired together the TSS coordinates of orthologous genes of two species in a single data column. An empty line separated each pair. The second data column contained a binary vector; ‘0’ for the coordinates of the first species and ‘1’ for that of other species. The two columns were scatter-plotted using ‘plot’ function with the *type* = *”l”* parameter on R-package. Wherever the split 3p21.31 clusters could not be mapped on a single scaffold, locations of genes flanking the breakpoint were assessed. The split occurred between Plxnb1 and Nme6 genes in hystricomorphs, and between Nme6 and Camp genes in sciuromorphs. If the genes flanking the breakpoint were on different scaffolds and their immediate neighbourhood had genes from elsewhere (from 3q and 19p13 in hystrico- and sciuromorphs, respectively), the 3p21.31 is considered as split.

### Hi-C analysis

SRA files of liver Hi-C datasets of mouse, dog, rabbit, and rhesus macaque were downloaded from Gene Expression Omnibus (GEO) with accession ID GSE65126 (Supplementary Table 1). Human liver Hi-C was downloaded from GSE58752 (Supplementary Table 1). NMR Hi-C data of embryonic fibroblasts was obtained from SRR8204318. SRA files were converted into fastq files using ‘fastq-dump’ of NCBI SRA Toolkit (https://github.com/ncbi/sra-tools) and processed using HiCUP (https://www.bioinformatics.babraham.ac.uk/projects/hicup/) with the parameter for restriction enzyme as ‘HindIII’ for human, mouse, dog, rabbit and macaque Hi-C data, and as ‘DpnII’ for NMR. The valid Hi-C pairs from HiCUP were built into contact maps, which were further normalised at 200 kb resolution using ‘simpleNorm’ method available in ‘Homer’ package (http://homer.ucsd.edu/homer/). Normalised contact maps of 3p21.31 and + /− 15MB neighbouring regions in human and other species were drawn using ‘heatmap’ function with scale ‘none’ on R. Snapshots (chr3:30–70 mb at 40 kb resolution) of Hi-C contact maps of other human tissues were directly obtained from PennState Hi-C browser (http://promoter.bx.psu.edu/hi-c/). The insulation domainograms were obtained using ‘insulation_domainogram’ function of GENOVA R-package (https://github.com/robinweide/GENOVA). The leading singular vectors of normalised Hi-C matrices were obtained using ‘svd’ function in R (https://www.rdocumentation.org/packages/base/versions/3.6.2/topics/svd). For the Hi-C matrices of various human tissues, the compartment scores (leading principal component) were directly obtained from Schmitt et al.^130^.

### Analysis of linkage disequilibrium (LD) and recombination rates

We used LD Score to analyse the linkage of alleles on human chr3. LD Score is the property of an SNP defined as the summation of Pearson’s correlation values *r*^2^, with all other SNPs in a 1 cM (centi Morgan) window. Human LD Score data for European-ancestry was obtained from download-server of Broad Institute (https://data.broadinstitute.org/mpg/snpsnap/database_download.html). LD scores were plotted using ‘geom_polygon’ function of ‘ggplot2’ R-package. *P* values for comparison between 3p21.31 (chr3:44–51 mb) and its neighbouring regions (upstream: 30–37 mb and downstream: 63–70 mb) were calculated using two-sided Mann–Whitney *U* tests on R. The recombination rates (at 5 mb resolution) of human and mouse genomes were obtained from Jensen-Seaman et al.^131^. The coordinates were lifted over to hg19 (human) and mm9 (mouse) assemblies using UCSC’s ‘liftover’ utility (https://genome.ucsc.edu/cgi-bin/hgLiftOver). The recombination rates were plotted without any further processing.

### Chromatin accessibility analysis

DNase-Seq data for 42 human and 27 mouse tissues were downloaded from ENCODE as bigWig files (Supplementary Table 1). These files were converted to ‘bedgraph’ files using the UCSC’s ‘bigWigToBedGraph’ utility. We binned DNase-seq signals at 1 Mb resolution and plotted mean (+/− 95% confidence intervals) values along 3p21.31 and adjacent loci. *P* values for comparison of DNase-seq enrichment at 3p21.31 region and neighbouring regions (25–35 mb upstream and 65–75 mb downstream) were calculated using two-sided Mann–Whitney *U* tests on R. The ATAC-seq data of NMR skin-fibroblast (Supplementary Table 1) was mapped along JH602043.1 scaffold (hetGla2) at 500 kb bins and smoothened using ‘running.mean’ function of ‘igraph’ R-package. *P* values for the comparisons of ATAC-seq signals at 0–4.6 mb and 37–42 mb regions, and at 37–42 mb and 73.4–78.8 mb regions of NMR scaffold were calculated using two-sided Mann–Whitney *U* tests on R.

### Replication timing analysis

The ‘bigwig’ files of Repli-chip data for four human cell-lines (GM06990, H9ES, HELA, and IMR90) and six mouse cell-lines (CH12, EPISC, ES, L1210F, MEFM, and MEL) were obtained from ENCODE (Supplementary Table 1) and converted to ‘bedgraph’ files using UCSC’s ‘bigWigToBedGraph’. We binned the Repli-chip signals at 1 Mb resolution and plotted mean (+/− 95% confidence intervals) values of Repli-chip enrichment along human chr3 and mouse chr9. *P* values were calculated using the same approach as in preceeding section.

### Gene expression, co-expression and divergence analyses

We calculated RPKM values from RNA-seq reads of the skin, heart, ovary, testis, liver, kidney and thymus tissues of adult NMR, guinea pig, human and mouse (Supplementary Table 1). For woodchuck, we obtained the data for the kidney, spleen and liver of adults (Supplementary Table 1). The datasets were quantile-normalised across tissues and species using ‘normalise.quantiles’ function of ‘preprocessCore’ R-package (https://rdrr.io/bioc/preprocessCore/) for the pair-wise comparisons of the expression levels. Median expression values across tissues were plotted for human chr3, and for corresponding orthologues in mouse. For individual genes, the quantile-normalised values were plotted as boxplots using ‘geom_boxplot’ function of ‘ggplot2’ R-package. *P* values for differential gene expression were calculated using two-sided Mann–Whitney U tests on R. For co-expression analysis, we calculated Pearson’s correlation coefficients among all genes ordered along human chromosome 3 using ‘cor’ function in R. The gene order of human chr3 was considered as reference for all co-expression matrices. The correlation matrices were further smoothened using the equation *n*_*i,j*_ = *(2m*_*ij*_ + *m*_*i-1,j*_ + *m*_*i,j-1*_ + *m*_*i+1,j+1*_ + *m*_*i,j+1*_*)/6* in three sequential iterations, where *n* is the smoothened matrix, *m* is the original matrix and *i* and *j* are the row and column indexes of matrices. This equation was implemented using ‘matrixSmooth’ function of ‘oce’ R-package(https://rdrr.io/cran/oce/man/oce.html). We plotted co-expression heatmaps using ‘pheatmap’ package using ‘spectral’ colour brewing scheme. The leading singular vector (SV) of co-expression matrix was calculated using ‘svd’ function in R. Expression divergence (d) of a gene between two different species was calculated using *equation d*_*i*_ = *1- ρ(x*_*i*_*, y*_*i*_*)* where *x*_*i*_ *and y*_*i*_ are vectors of quantile-normalised expression values of gene *i* across multiple tissues of species *x* and *y*. The divergence values were smoothed using ‘running.mean’ function of ‘igraph’ R-package with binwidth=10.

### Analysis of conserved non-coding elements (CNEs)

Hg19 chromosomal cooridnates of mammalian CNEs present in 3p21.31 region were obtained from^132^ and lifted over to mouse (mm10) using ‘liftover’ (https://genome-store.ucsc.edu/) chains at 0.95 mapping coverage. The mouse-conserved CNEs were further lifted over to NMR (HetGla2), guinea pig (CavPor3), Woodchuck (GCA_901343595.1), rat (Rn6), and dog (CanFam3) using the same parameters. The CNEs that were partially or fully deleted in respective species were considered as lost cases in that species. The proportions of lost cases were calculated for each species.

### Gene ontology (GO)

All gene ontology analyses were performed using ToppGene Suite (https://toppgene.cchmc.org/) with Benjamini–Hochberg correction of *P* values.

### GC content along NMR and alpine marmot scaffolds

The genome sequences (fasta) of NMR and alpine marmot were downloaded from the UCSC genome browser, and NCBI respectively. The DNA sequences of NMR scaffold JH602043.1 and alpine marmot scaffold CZRN01000025.1 were fetched and the percentage of ‘G + C’ nucleotides in 100-kb bins were calculated. The values were smoothened using ‘running.mean’ function of ‘igraph’ R-package with binwidth=10. *P* values for the difference in GC contents between 0–4.6 mb and 37–42 mb, and between 37–42 mb and 73.4–78.8 mb regions were calculated using two-sided Mann–Whitney *U* tests on R. For alpine marmot, the size of split 3p21.31 sub-cluster and the 19p13.11 locus were comparable, and therefore GC% values in the whole regions were used to calculate *p* values using two-sided Mann–Whitney *U* tests.

### LaminB1 enrichment analysis

LaminB1 ChIP-Seq (MEF) data of mouse orthologous regions of NMR scaffold JH602043.1 and alpine marmot scaffold CZRN01000025.1 were taken from GSE17051 (Supplementary Table 1). Smoothened values were plotted along scaffolds. *P* values for comparison of enrichment at 0–4.6 mb and 37–42 mb, and at 37–42 mb and 73.4–78.8 mb NMR orthologous regions in mouse were calculated using two-sided Mann–Whitney *U* tests. Like in the case of GC content, the LaminB1 enrichment values in the whole of split 3p21.31 and 19p13.11 regions were used to calculate *P* values using two-sided Mann–Whitney *U* tests.

### Evolutionary rate calculations

Evolutionary rates of lineage-specific expression divergence for the genes within 3p21.31 locus and upstream/downstream loci (50 genes each side) were calculated and tested for significance using ‘TreeExp’ R-package (https://github.com/hr1912/TreeExp). NMR and woodchuck were taken as ‘test’, mouse was considered as ‘reference’, and human served as ‘out-group’ in the analysis. Body mass rates for 312 rodent species were calculated using ‘BAMM’ package (http://bamm-project.org/). We plotted body mass rate as a function of evolutionary time using ‘BAMMtools’ R-package (https://cran.r-project.org/web/packages/BAMMtools/index.html). Phylogenetic tree (Newick format) was obtained from TimeTree (http://www.timetree.org/) (Supplementary Table 1). Body mass and activity data of extant rodents and their inferred ancestors were superimposed onto phylogenetic trees using ‘contMap’ function of ‘phytools’ R-package (http://www.phytools.org/). Pagel’s **λ** was calculated using ‘phyloSig’ function of ‘phytools’. Density plots of body mass for the rodent suborders were made using ‘geom_density’ function of ‘ggplot2’ R-package.

### Statistical tests

We mostly used two-sided Mann–Whitney *U* test and two-tailed *t* test to assess the statistical significance. Effect sizes were calculated using Cohen’s *d* and *η*^2^ for *t* tests and Mann–Whitney *U* tests using Eqs. (1) and (2), respectively.

1 $${Cohen}^\prime {s}\;d = \frac{{m1 - m2}}{{S_{pooled}}}$$

Where, m1 and m2 are mean of sample 1 and 2, respectively, and S_pooled_ is the pooled standard deviation of two samples.

2 $$\eta ^2 = \frac{{z^2}}{N}$$

where, $$z = \frac{{U - m_U}}{{\sigma _U}}, m_U = \frac{{n1.n2}}{2}$$ and $$\sigma _U = \sqrt {\frac{{n1.n2.(n1 + n2 + 1)}}{{12}}}$$

n1 and n2 are sizes of samples 1 and 2. *U* is the Mann–Whitney *U* value. m_U_ is the mean *U* value. z is the *Z* score of *U* value calculated as above.

### Reporting summary

Further information on research design is available in the Nature Research Reporting Summary linked to this article.

## Supplementary information

Supplementary Information

Supplementary Data 1

Reporting Summary

## Data Availability

The mapped datasets are available as online Supplementary Data.

## Code availability

The custom scripts used for data analyses are available on request.
