# Supplementary material for: Convergent evolution of a genomic rearrangement may explain cancer resistance in hystrico- and sciuromorpha rodents
Source: NPJ Aging Mech Dis. 2021 Sep 1;7:20. doi: 10.1038/s41514-021-00072-9 (PMC8410860; doi:10.1038/s41514-021-00072-9)
Supplement: Supplementary file 1 — Supplementary Information [file 41514_2021_72_MOESM1_ESM.pdf]

**Supplementary Information**

# Convergent evolution of a genomic rearrangement may explain cancer resistance in hystrico- and sciuromorpha rodents

Yachna Jain[1], Keerthivasan Raanin Chandradoss[1], Anjoom AV[1], Jui Bhattacharya, Mohan Lal, Meenakshi Bagadia, Harpreet Singh, Kuljeet Singh Sandhu[*]

Department of Biological Sciences
Indian Institute of Science Education and Research (IISER) – Mohali
Knowledge City, Sector 81, SAS Nagar 140306, Punjab, India.

[1]Equal contribution
[*]Correspondence to sandhuks@iisermohali.ac.in

**Supplementary Table 1.** Details of the datasets used in the study

| Analysis | Data-type / tool | Species | Tissue | Database | Accession / link |
|---|---|---|---|---|---|
| Gene expression | RNA-Seq | human | Thymus | GEO ENCODE | GSM2935638 |
| | | | Skin | | ENCFF549LHP |
| | | | Ovary | | ENCFF479CKA |
| | | | Kidney | | ENCFF251XMZ |
| | | | Adrenal | | ENCFF480JAT |
| | | | Heart | | ENCFF205LNA |
| | | | Testis | | ENCFF662BIH |
| | | | Liver | | ENCFF039WAC |
| | | mouse | Skin | | GSM1606093 |
| | | | Thymus | | ENCFF074ZUD |
| | | | Ovary | | ENCFF097YQV |
| | | | Kidney | | ENCFF167GTO |
| | | | Adrenal | | ENCFF916CFV |
| | | | Heart | | ENCFF456GZP |
| | | | Testis | | ENCFF456PSH |
| | | | Liver | | ENCFF880OSE |
| | | NMR | Skin | | GSE98719 |
| | | | Thymus | | |
| | | | Ovary | | |
| | | | Kidney | | |
| | | | Adrenal | | |
| | | | Heart | | |
| | | | Testis | | |
| | | | Liver | | |
| | | Guinea pig | Skin | | |
| | | | Thymus | | |
| | | | Ovary | | |
| | | | Kidney | | |
| | | | Adrenal | | |
| | | | Heart | | |
| | | | Testis | | |
| | | | Liver | | |
| | Array | Woodchuck | Liver, Spleen, Kidney | | GPL15354 |
| Spatial proximity | Hi-C | Human | Aorta | PennState Hi-C browser | Leung et al (2015) Schmitt et al (2016) |
| | | | Liver | | |
| | | | Adrenal | | |
| | | | Bladder | | |
| | | | Psoas | | |
| | | | Pancreas | | |
| | | | Spleen | | |
| | | Mouse | Liver | GEO | GSE58752 GSE65126 |
| | | Monkey | | | |
| | | Dog | | | |
| | | Rabbit | | | |

| | | | | | |
|---|---|---|---|---|---|
| | | NMR | Embryonic fibroblasts | SRA | SRR8204318 |
| Chromatin accessibility | ATAC-Seq | NMR | Skin fibroblast | GEO | GSE83585 |
| | DNase-Seq | Human | Body of pancreas | ENCODE | ENCFF095KFF |
| | | | Breast epithelium | | ENCFF322ALX |
| | | | Upper lobe of left lung | | ENCFF414NAA |
| | | | Tibial artery | | ENCFF367MHB |
| | | | Gastrocnemius medialis | | ENCFF082TEM |
| | | | Ovary | | ENCFF338CIA |
| | | | Thyroid gland | | ENCFF126DFR |
| | | | Esophagus muscularis mucosa | | ENCFF951HWM |
| | | | Coronary artery | | ENCFF871GME |
| | | | Prostate gland | | ENCFF069GRI |
| | | | Stomach | | ENCFF922TBR |
| | | | Small intestine | | ENCFF619LIB |
| | | | Lower leg skin | | ENCFF561DJM |
| | | | Pancreas | | ENCFF230OUF |
| | | | Omental fat pad | | ENCFF037ZSW |
| | | | Tibial nerve | | ENCFF094FZF |
| | | | Esophagus squamous epithelium | | ENCFF376RIO |
| | | | Ascending aorta | | ENCFF233ORX |
| | | | Vagina | | ENCFF154NSS |
| | | | Transverse colon | | ENCFF413EKZ |
| | | | Sigmoid colon | | ENCFF410TTD |
| | | | Right lobe of liver | | ENCFF462ZLK |
| | | | Spleen | | ENCFF047HXB |
| | | | Uterus | | ENCFF037DUA |
| | | | Testis | | ENCFF068YUS |
| | | | Heart left ventricle | | ENCFF678EIG |
| | | | Adrenal gland | | ENCFF150WZO |
| | | | Frontal cortex | | ENCFF110QGM |
| | | | Globus pallidus | | ENCFF225FYX |
| | | | Pons | | ENCFF210SQU |
| | | | Midbrain | | ENCFF547ZQF |
| | | | Heart | | ENCFF303XQF |
| | | | Cerebellar cortex | | ENCFF564DYF |
| | | | Putamen | | ENCFF681JFJ |
| | | | Psoas muscle | | ENCFF846FZJ |
| | | | Inferior parietal cortex | | ENCFF848BGL |
| | | | Occipital lobe | | ENCFF568EAH |
| | | | Superior temporal gyrus | | ENCFF029ZOX |

| | | | | | |
|---|---|---|---|---|---|
| | | | Medulla oblongata | | ENCFF151SBP |
| | | | Middle frontal gyrus | | ENCFF751LHL |
| | | | Caudate nucleus | | ENCFF498QFP |
| | | Mouse | Lung | | ENCFF043UNG |
| | | | Forelimb bud | | ENCFF837OUY |
| | | | Hindbrain | | ENCFF943PHW |
| | | | Limb | | ENCFF641OPE |
| | | | Brain | | ENCFF992QCB |
| | | | Forebrain | | ENCFF684BQC |
| | | | Embryo | | ENCFF627IDE |
| | | | Retina | | ENCFF908NIP |
| | | | Neural tube | | ENCFF649UQO |
| | | | Embryonic facial prominence | | ENCFF311WRA |
| | | | Liver | | ENCFF395XSI |
| | | | Midbrain | | ENCFF942RUO |
| | | | Heart | | ENCFF773NKY |
| | | | Kidney | | ENCFF299KAN |
| | | | Hindlimb bud | | ENCFF037VPV |
| | | | stomach | | ENCFF773UVA |
| | | | Thymus | | ENCFF125IRZ |
| | | | Gonadal fat pad | | ENCFF090NJT |
| | | | Large intestine | | ENCFF650OFZ |
| | | | Yolk sac | | ENCFF353HPD |
| | | | Cerebellum | | ENCFF474GND |
| | | | Intestine | | ENCFF424BHU |
| | | | Spleen | | ENCFF546FZT |
| | | | Telencephalon | | ENCFF238ZRM |
| | | | Fat pad | | ENCFF243REK |
| | | | Mesoderm | | ENCFF673VVB |
| | | | Skeletal muscle tissue | | ENCFF731YAA |
| | | | | | |
| TSGs & POGs | | Human | | CancerMine | http://bionlp.bcgsc.ca/cancermine/ |
| GC content | | NMR | | UCSC | https://genome.ucsc.edu/ |
| Linkage disequilibrium | | Human | | Broad Institute FTP server | http://homer.ucsd.edu/homer/interactions/ |
| Lamin-B1 enrichment | ChIP-Seq | Mouse | ESC | GEO | GSE17051 |
| Replication timing | Repli-chip | Human | GM06990 | ENCODE | https://hgdownload-test.gi.ucsc.edu/goldenPath/hg19/encodeDCC/wgEncodeFsuRepliChip/release1/ |
| | | | H9ES | | |
| | | | Hela | | |
| | | | IMR90 | | |
| | | Mouse | CH12 | | http://hgdownload.cse.ucsc.edu/goldenpath/mm9/encodeDCC/wgEncodeFsuRepliChip/ |
| | | | EPISC | | |
| | | | ESC | | |
| | | | L1210F | | |
| | | | MEFM | | |
| | | | MEL | | |

| | | | | | |
|---|---|---|---|---|---|
| UV transmission | 50% T | 38 mammals | Eye lens | | Douglas and Jeffery (2014). https://doi.org/10.1098/rspb.2013.2995 |
| Recombination rates | | Human and mouse | | - | Jensen-Seaman et al, 2004 |
| Temporal activity data | | >4000 species | | AnAge | https://genomics.senescence.info/species/stats.php |
| | | | | | |
| Hi-C processing | SRA Toolkit | | | | https://www.ncbi.nlm.nih.gov/sra/docs/toolkitsoft/ |
| | HiCUP | | | | https://www.bioinformatics.babraham.ac.uk/projects/hicup/ |
| | Homer | | | | http://homer.ucsd.edu/homer/interactions/ |
| | PennState Hi-C browser | | | | http://promoter.bx.psu.edu/hi-c/ |
| CNVs, gene expression across cancers | Xena browser | Human | | | https://xenabrowser.net/ |
| Orthologous chr/scaffold coordinates | UCSC Liftover | | | | https://genome.ucsc.edu/cgi-bin/hgLiftOver |
| Gene ontology | ToppGene suite | | | | https://toppgene.cchmc.org/ |
| Genome browsers | UCSC genome browser | | | | https://genome.ucsc.edu/ |
| | Ensembl genome browser | | | | https://asia.ensembl.org/index.html |
| Analysis of body mass and max. longevity | TimeTree | Myomorpha, Hystricomorpha & Sciuromorpha | | | http://www.timetree.org/ |
| | Phytools | | | | http://www.phytools.org/eqg2015/asr.html |
| | AnAge database | | | | https://genomics.senescence.info/species/ |
| Statistics | R | | | | https://www.r-project.org/ |
| Visualization | ggplot2 | | | | https://ggplot2.tidyverse.org/ |

**Supplementary Table 2.** Distances between NME6 and PLXNB1 genes in genomes of human, myomorphs, sciuromorphs and hystricomorphs. In hystricomorphs, the genes were present in different scaffolds. To calculate the NME6-PLXNB1 distances, the scaffolds were joined in all possible orientations (1 convergent, 1 divergent and 2 tandem orientations), and the smallest distance of all combinations (the worst case scenario) was considered.

| **Species** | **Chr/Scaffold** | **Plxnb1-Nme6 distance (Mb)** | **Chr/Scaffold** | **Nme6-Camp distance (Mb)** |
|---|---|---|---|---|
| Human | Chr3 | 0.113132 | Chr3 | 0.067375 |
| **Myomorphs** | | | | |
| Mouse | Chr9 | 0.73736 | Chr9 | 0.014630 |
| Rat | Chr8 | 0.084243 | Chr8 | 0.009602 |
| Mongolian gerbil | NHTI01000042.1 | 0.08431 | NHTI01000042.1 | 0.010237 |
| Golden hamster | KB708177.1 | 0.095007 | KB708177.1 | 0.014740 |
| Blind mole rat | KL204508.1 | 0.537969 | KL204508.1 | 0.044576 |
| Kangaroo rat | KN672453.1 | 0.045737 | KN672453.1 | 0.005384 |
| **Sciuromorphs** | | | | **Min. of all orientations** |
| Squirrel | JH393339.1 | 0.111261 | Different scaffolds | 1.800195 |
| Alpine Marmot | CZRN01000020.1 | 0.146779 | Different scaffolds | 8.122866 |
| Arctic ground squirrel | Different scaffolds | 0.155398 | Different scaffolds | 2.557477 |
| Daurian squirrel | PLXNB1 ortholog not found | | Different scaffolds | 0.388004 |
| **Hystricomorphs** | | | | |
| NMR | JH602043.1 (hetGla2 assembly) | 71.045 | JH602043.1 | 0.015136 |
| | RPGA1000013.1(GCA_014060925.1 assembly) | 78.407 | RPGA1000013.1(GCA_014060925.1 assembly) | 0.015071 |
| **Other hystricomorphs** | | **Min. of all orientations** | | |
| Chinchilla | Different scaffolds | 6.413811 | CAMP Ortholog not found | |
| Brazillian guinea pig | Different scaffolds | 36.147762 | AVPZ01000778.1 | 0.014144 |
| Damara mole rat | Different scaffolds | 5.109729 | KN122104.1 | 0.011282 |
| Degu | Different scaffolds | 2.284109 | Different scaffolds | 2.175631 |
| North American porcupine | Different scaffolds | 1.281664 | ML655359.1 | 0.010105 |
| Domestic guinea pig | Different scaffolds | 36.149925 | DS562862.1 | 0.015158 |

**Supplementary Table 3.** List of genes, located in 3p21-22 (sea green), 3q (red), 19p13.11 (orange), and other (black) loci, that are associated with evolutionarily diverged traits in hystrico- and sciuromorphs

| Function | Genes | Cytoband | Role |
|---|---|---|---|
| Negative regulation of growth | SEMA3F,HYAL1,CCR2, SEMA3B,NME6,HYAL 2,IP6K2, SETD2 | 3p21.31 | Growth and tumour suppression |
| UV response | HYAL1, HYAL2, HYAL3, TMEM161A | 3p21.31, 19p13.11 | Cancer, UV-response, vision, skin elasticity |
| Hyaluronan binding | HAPLN4, NCAN | 19p13.11 | hibernation |
| Inflammatory response | XCR1,TREX1,IMPDH2, HYAL1,CCR9,TRAIP,T DGF1,CCR2,MST1R,H YAL3,CCR1,CCR3,SLC 26A6,HYAL2,IP6K2 | 3p21.31 | Cancer, wound healing |
| DNA metabolism | EREG,AREG,CXCL8,PA RM1 | 4q13 (NMR-4) | Genome maintenance |
| Thermoregulation | PTH1R | 3p21.31 | Poikilothermism, hibernation |
| Hypoxia tolerance | UQCRC1 | 3p21.31 | Sub-terranean habitat, hibernation |
| Vitamin-A metabolism | GRM2, RBP1, RBP2 | 3p21.2, 3q23 | Vision, hibernation, |
| Enamel mineralization | ENAM, AMTN | 20p13(GP-2) | Grinding |
| Mating behaviour | OXT,AVP, | 3q(GP-1) | Diverse mating behaviour |
| Amino Acid metabolism | GLYCTK, ALAS1, GRM2 | 3p21.2 | hibernation |
| Regulation of telomere maintenance | PARP3, CERS1, XRN1, ATR, STAG1, CEP63 | 3p21.2, 19p13.11, 3q | Aging, cancer-resistance |
| Atrial blood pressure | MANF | 3p21.2 | Hibernation |
| Regulation of kinase activity | CISH, DUSP7, MAPKAPK3, BORCS8, MEF2B, DOCK3 | 3p21.2,19p13.11 | Hibernation |
| Blood coagulation | FGA,FGB,FGG | 4q28(GP-3) | Wound healing |
| Lipid homeostasis | SCAP, ACAD11, TM6S F2, PLSCR gene-family, ESYT3, SLCO2A1, AGTR1, | 3q22/23, 19p13.11, 3p21.31, 3p22 | Hibernation |
| Calmodulin binding | IQCF1, IQCF2, IQCF 3, IQCF4, IQCF5, IQ CF6 | 3p21.2 | Hibernation |
| Eye development | SOX2,PCYT1A, SCAP, DHX30, CDC25A | 3q(NMR-5), 3p21.31 | Vision |
| Matrix (other than HA related) | COL6A6, PCOLCE2, PLOD2, PLXNB1, CSPG5, CILP2 | 3q22.1, 3p21.31, 19p13.11 | Wound healing, aging, cancer, skin etc. |

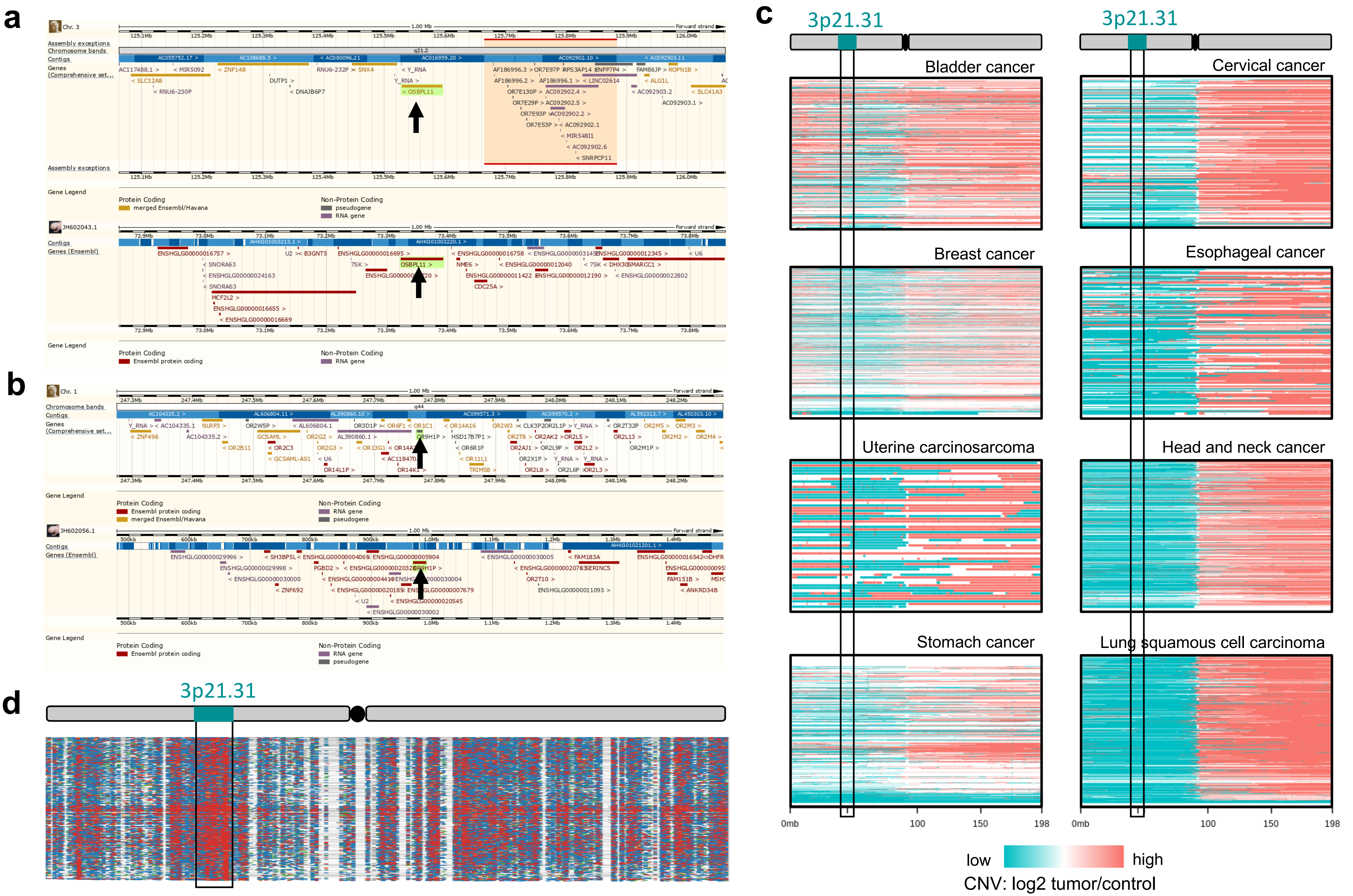


**Supplementary Figure 1.** (a-b) Single gene repositioning events between Human (top panel) and NMR (bottom panel). The OSBPL11 and OR9H1P genes are indicated by arrow-heads. Comparing the immediate neighbourhood of these genes in Human and NMR negates the possibility of translocation or inversion. OSBPL11 is adjacent to a region marked as "assembly exceptions" (highlighted in red), implying the possibility of erroneous sequence mapping. OR9H1P lacked introns and is annotated as pseudogene, hinting at differential retro-transposition of the gene in human and NMR. (c) Amplification and deletion of various chr3 regions across different cancers in human. (d) Frequency of somatic point mutations in TCGA cohort. Data was directly pulled down from UCSC Xena browser. Red: deleterious mutations; blue: missense mutations.

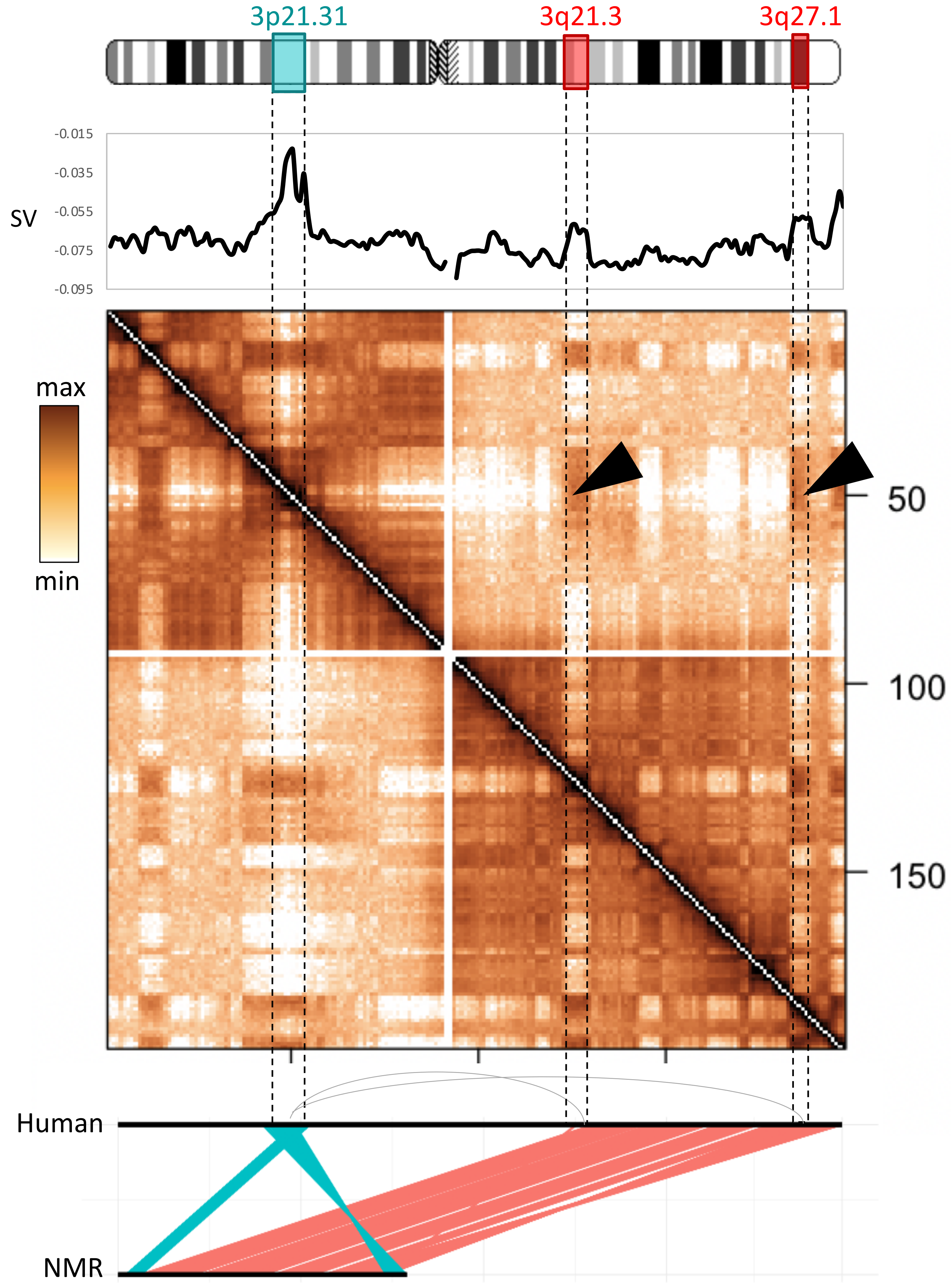


**Supplementary Figure 2.** Hi-C contact map of entire chr3 of human (liver, 1Mb resolution), aligned onto the map of human-NMR genomic rearrangement. The top panel shows chr-3 ideogram followed by the leading singular vector of normalized Hi-C contact matrix. Arrow-heads highlight the regions that show high frequency of contacts with 3p21.31 locus. These regions coincide with the human-NMR breakpoints on 3q arm.
